# How Much Energy Storage can We Afford? On the Need for a Sunflower Society, Aligning Demand with Renewable Supply

**DOI:** 10.1007/s41247-022-00097-y

**Published:** 2022-04-28

**Authors:** Harald Desing, Rolf Widmer

**Affiliations:** Empa - Swiss Federal Laboratories for Material Science and Technology, Lerchenfeldstrasse 5, 9014 St. Gallen, Switzerland

**Keywords:** Energy transition, Energy storage, Climate risks, Climate crisis, Energy demand management

## Abstract

The worsening climate crisis impels society to accelerate climate action. The attainable speed of the energy transition is ultimately limited by the available energy to build the replacing renewable infrastructures. Decarbonizing the energy system by replacing dispatchable fossil with variable renewable power requires energy storage to match supply with demand. Current storage technologies are energetically expensive to build and operate, thus the demand for storage shapes the fastest possible transition and the probability to exceed 1.5 °C heating. This study explores and quantifies the effect of demanded storage and its technological progress on the fastest possible transition constrained only by energy. The simulation results using three exemplary storage technologies show that storage substantially delays the transition and increases the probability to exceed 1.5 °C heating. Technological progress, if materialized fast, can reduce energy costs of storage; however, storage demand remains a critical driver for climate risks. Consequently, minimizing storage demand through a supply-driven power system effectively reduces climate risks—a paradigm shift towards a solar-aligned “sunflower society”.

**Supplementary Information:**

The online version contains supplementary material available at 10.1007/s41247-022-00097-y.

## Introduction

With the climate crisis unfolding (WMO 2021), the urgency for climate action is coming to the fore (IPCC 2022). Peak heating of 1.5 °C is considered the vital threshold for averting changes in the Earth system dangerous for ecosystems and human prosperity (Lenton et al. 2019; Hoegh-Guldberg et al. 2019; IPCC 2018). Yet, energy transition pathways are designed so that they will exceed 1.5 °C peak heating with a chance of $>\,40\%$ (Desing and Widmer 2021; IPCC 2018) or even allow for a “slight” overshoot (IPCC 2022). In line with the precautionary principle, climate action should be designed for minimizing climate risks, most notably by limiting cumulative CO_2_ emissions and the probability to exceed 1.5 °C (Desing and Widmer 2021). Regardless which level of climate risks is considered “safe”, continued inadequate climate action necessitates an acceleration of the energy transition to meet any such target. Accelerating the energy transition, however, faces limits. Most importantly, it requires energy to build the renewable infrastructure. During the transition, this energy has to be provided in addition to the energy demand of society. As shown previously (Desing and Widmer 2021), the fastest possible energy transition—i.e. a complete replacement of the current fossil with a solar energy supply system without energy storage and constrained only by energy—can be achieved by increasing fossil power and emissions with the sole purpose of building solar infrastructure. The resulting very short transition time can substantially reduce cumulative CO_2_ emission and thus the probability of exceeding 1.5 °C.

Given the variability of solar irradiance, some energy storage will be required in a 100% solar-powered supply system. Several studies identify energy storage as critically important to the energy transition, either because of its energy and material intensity (Barnhart et al. 2013; Barnhart and Benson 2013) or its costs (Brown et al. 2018). The practical viability of 100% renewable energy systems is controversially discussed mainly because of the extensive storage requirements to ensure supply and grid stability (Jacobson et al. 2017; Brown et al. 2018). Storage demand is increasing non-linearly with decarbonization because of lacking dispatchable fossil power to balance variable renewable supply (Victoria et al. 2019). Consequently, most transition pathways project continued fossil emissions beyond 2100 and thus have to rely on negative emissions to reach “net zero” (van Vuuren et al. 2018).

Adding energy storage to the fastest possible and complete transition from fossil to solar will increase cumulative CO_2_ emissions and therewith the probability to exceed 1.5 °C, as the additional energy necessary to build and operate storage will delay the transition. Due to the variability of solar supply, which is asynchronous to power demand for some uses (e.g. lighting), energy storage is inevitable in a solar-powered society. The demand for storage, however, very much depends on how well power demand is synchronized with solar supply. It can be aligned e.g. via demand flexibility or sector coupling as well as interconnection of larger areas (Brown et al. 2018). Other studies estimate storage demand based on matching supply with demand scenarios in high temporal and spatial resolution (Bogdanov et al. 2019; Pleßmann et al. 2014); however, they are commonly aiming for finding a cost optimal rather than a climate optimal solution.

This study explores the implications of energy storage on fast and complete energy transitions when only constrained by energy. The aim is to quantify the effect of adding energy storage on the probability to exceed 1.5 °C heating. It does not, however, attempt to evaluate demand and supply alignment in great detail. The potential of regional or trans-national grids, sector coupling or dispatchable renewables on reducing storage demand is outside the scope of this study. Since existing renewable energy installations other than solar are already close to or even beyond their potential within planetary boundaries (Desing et al. 2019), the development of solar is taken as the replacement of current fossil infrastructure. The already installed renewable energy capacity, i.e. mainly in hydro, biomass and wind (IEA 2020), is assumed to remain constant during the transition.

This paper explores the influence of energy storage on the fastest possible energy transition (Desing and Widmer 2021) using different exemplary energy storage options (“Energy Storage Options and Their Energetic Costs” section). This is done by expanding the energy transition model from Desing and Widmer (2021) (“Transition Model” section) with storage demand across a wide range of feasible parameters (“Storage Demand” section). The influence of storage on fastest possible energy transitions is discussed in “Results” section, its consequences and implications for society in “Discussion” and “Conclusions and Implications” sections.

## Materials and Methods

The purpose-built model (Desing and Widmer 2021) to explore the relation between physically attainable transition speed and cumulative CO_2_ emissions was expanded here (“Transition Model” section) to investigate the influence of energy investments in storage infrastructures (“Energy Storage Options and Their Energetic Costs” section) necessary to satisfy demand (“Storage Demand” section). Each of the three exemplary storage options is applied independently as different scenarios. Direct solar energy conversion on the surface of the already built environment is considered as the principal renewable energy resource to replace fossil energy supply, as it has by far the largest unused sustainable potential today (Desing et al. 2019) and is currently growing fastest (Desing and Widmer 2021; British Petroleum 2020). It furthermore does not require any additional land transformation when installed on currently existing rooftops, facades, and other infrastructure areas. Today’s renewable part of the energy system is assumed to stay constant and is excluded from the model.

Energy demand currently provided by the fossil power system, which in the model is replaced by solar supply, is taken as $P_{\rm demand}=6$ TW (IEA 2020, 2021; British Petroleum 2020) in electric energy equivalents, i.e. as if they were already converted to electricity for final use (Desing et al. 2019; Desing and Widmer 2021). Thus, solar power supply has to replace all final power demand of same magnitude. The transition simulated in this study is assumed to start on 1 January 2023 and annual CO_2_ emissions as well as fossil power supply is held constant until the end of 2022. Land use-related CO_2_ emissions are assumed to reduce to net zero in 2023.

The probability for exceeding 1.5 °C is calculated using the remaining carbon budget values provided by the sixth assessment report of IPCC (IPCC 2021). A log-normal distribution is fitted to the data points provided ($R^2=0.99$). The probability distribution got narrower in comparison with the former special report on 1.5 °C (IPCC 2018), which was used in Desing and Widmer (2021) (see Fig. S1 for comparison).

### Storage Demand

Storage demand is modelled with two parameters: independence time $\Delta t_i$, describing the size of installed storage capacity and therewith energy investments to built the storage infrastructure; and fraction of demand stored $\phi$, determining the actual amount of final demand provided through storage and therewith energy losses in storage operations.

Storage needs to be able to provide the required annual average power demand $P_{\rm demand}$, enlarged by storage output losses ($\frac{1}{\eta_{\rm out}}$), throughout a chosen independence time $\Delta t_{i}$. This requires a certain amount of storage capacity $E_{\rm storage\, capacity}$:

1 $$\begin{aligned} \Delta t_{i}= & {} \frac{E_{\rm storage \, capacity}\cdot \eta_{\rm out}}{P_{\rm demand}} \end{aligned}$$

**Fig. 1:**
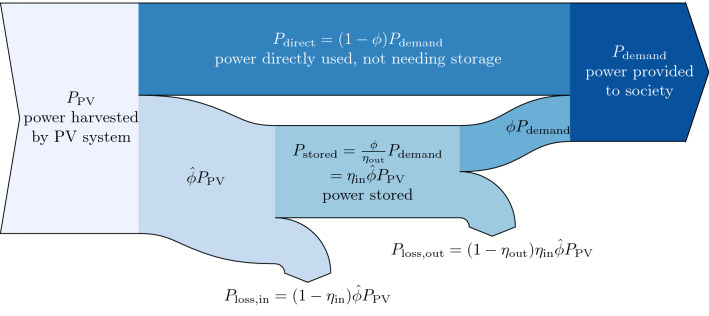
Energy flow from solar supply ($P_{\rm PV}$) through storage to satisfy demand by society ($P_{\rm demand}$). The fraction $\phi$ of demand is supplied through storage, while the remainder is supplied directly from PV

This value can be interpreted as the time throughout which the entire demand by society can be supplied through storage alone. The output efficiency $\eta_{\rm out}$ determines how much larger storage capacity needs to be to store demand plus storage output losses (Fig. 1).

For levelling out diurnal solar supply and satisfy constant demand, an independence time of $\Delta t_i = 14.5\,\text{h} = 0.0017\,\text{a}$ (Sect. S2) is required. Cloudy and windless periods increase the independence time up to the order of days, whereas seasonal variations of solar availability increase the required independence time up to months. However, as most of the world’s population is currently living around the equator (“sun belt”) where seasonal variations are low (Victoria et al. 2021), the global need for storage capacity will likely not exceed $\Delta t_i < 0.1\,\text{a}$. In the following, the independence time is varied within the range from no storage $\Delta t_i = 0\,\text{a}$ to a maximum of $\Delta t_i = 0.1\,\text{a}.$

**Fig. 2:**
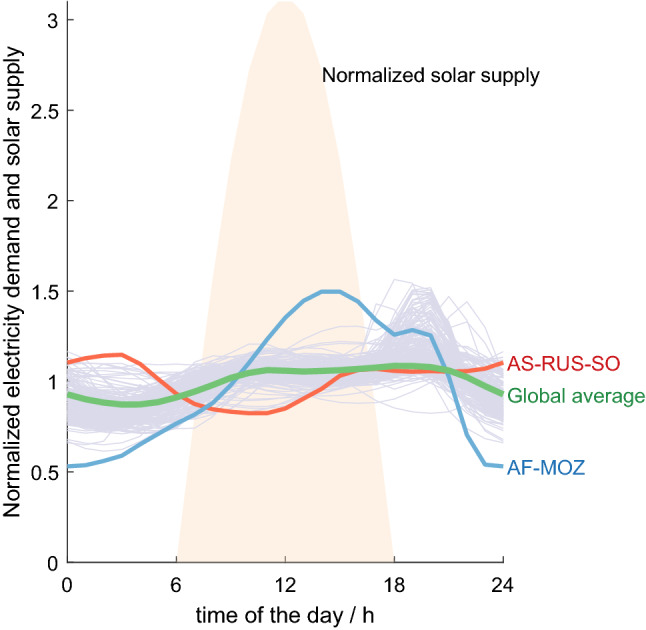
Annual average electricity demand profiles for 270 world regions (Brinkerink et al. 2021) in comparison with normalized solar supply. All lines are normalized so that the area under each curve is equal and shifted to local time. Global average highlighted in green; Mozambique (AF-MOZ) has the lowest storage demand (blue), while in the south of Russia (AS-RUS-SO), demand is most unaligned to solar supply, requiring most storage (red) (Color figure online)

Storage losses depend on the round-trip efficiency $\eta$ and the fraction of demand actually stored $\phi$. Round-trip efficiency is a technological parameter (Table S3) and can be split into the input efficiency $\eta_{\rm in}$ (“charging”) and the output efficiency $\eta_{\rm out}$ (“discharging”).

2 $$\begin{aligned} \eta& \qquad = \qquad \frac{P_{\rm out}}{P_{\rm in}} = \eta_{\rm in}\cdot \eta_{\rm out} \\ P_{\rm out} &\qquad \dots \qquad \text{average \; power \; output \; from \;storage } \\ P_{\rm in}&\qquad \dots \qquad \text{ average \; power \; input \;necessary \; to \; provide \; the \; output } \end{aligned}$$

The storage fraction $\phi$ determines how much of the annual average final demand is actually provided through storage. Assuming a constant average demand over the course of a day, there is a need to store $\phi_{\rm d,max}=0.58$ of the daily energy demand (Sect. S2). As energy demand is generally higher during the day, this can be considered the upper limit of daily storage requirements. Current electricity use patterns (Brinkerink et al. 2021) show globally somewhat less storage demand $\phi_{\rm d,global}=0.54$ in 2015 (Fig. 2). The country with the electricity demand pattern best aligned to solar supply is Mozambique ($\phi_{\rm d,MOZ}=0.45$), while the southern region of Russia has the highest demand for storage ($\phi_{\rm daily,RUS-SO}=0.59$). Today, constant demand profiles are beneficial for thermal power plant operations, which is encouraged in many regions with lower electricity tariffs during the night, providing an incentive to shift energy demand from day to night. However, keeping up this demand patterns implies a large storage fraction $\phi$ and creates a challenge for the transition to a solar-powered society.

Mobility demand peaks in the mornings and evenings when solar supply is low and is low during the night (Brown et al. 2018). For disconnected mobility (e.g. battery electric vehicles), charging profiles are delayed by average trip time, while energy demand for grid-connected mobility (e.g. trolley bus, train) mobility and energy demand are simultaneous. Electrifying mobility with current demand patterns requires a storage fraction of $\phi_{\rm mobility} = 0.39$, which is somewhat lower than for current electricity demand patterns (Fig. S5).

For seasonal storage, additional energy has to be stored to level out beyond daily variations (periods of cloud cover, annual variations in higher latitudes) and fulfil demand during seasons with low irradiation and high demand for space heating. In contrast, heat demand is anti-cyclical to seasonal solar supply in Europe (Brown et al. 2018), which may increase global $\Delta t_i$ and $\phi$. Even though the majority of the world’s population live within the sun belt with low seasonal variations, Europe and North America are responsible for a high share in energy demand. As an upper limit, seasonal storage fraction is maximally $\phi_{\rm a,max}=0.2$. Consequently, the fraction of daily average demand, which needs to be provided through storage, is simulated in the range between $\phi =[0.2,0.8]$.

Storage fraction is limited by independence time smaller than the minimum charge/discharge time $\Delta t_{\rm charge}$. There needs to be enough storage capacity available to actually be able to store the required storage fraction.

3 $$\begin{aligned} \phi< & {} \frac{P_{\rm storage,nom}\eta_{\rm out}}{P_{\rm demand}} = \frac{E_{\rm storage\; capacity}\eta_{\rm out}}{\Delta t_{\rm charge} P_{\rm demand}} = \frac{\Delta t_i}{\Delta t_{\rm charge}} \end{aligned}$$

4 $$\begin{aligned} \Delta t_{\rm charge}= & {} \frac{E_{\rm storage capacity}}{P_{\rm storage,nom}} \end{aligned}$$

As $\Delta t_{\rm charge}$, which can be also interpreted as the capacity-to-power ratio, is usually smaller than the necessary independence time (Bogdanov et al. 2019), this dependence is omitted in the further calculations.

In comparison with other studies, Pleßmann et al. (2014) find the storage demand for 100% renewable electricity without demand flexibility and electrification of heat and transport with a storage fraction $\phi =0.5$ and independence time $\Delta t_i = 0.06\,\text{a}$ (Table S1). Bogdanov et al. (2019) provide a scenario for storage demand after allowing for demand flexibility and sector coupling, reducing the need for storage compared to the current demand pattern to minimize system costs. This scenario reduces storage fraction to $\phi =0.28$ and independence time to $\Delta t_i = 0.018\,\text{a}$ (Table S2).

### Energy Storage Options and Their Energetic Costs

Many different technologies to store energy are in use or under development (Letcher 2016; Koohi-Fayegh 2020). They differ in the physical principles, in energetic performance and resource demand. Energy storage options require both energy to build (embodied energy) and to operate (storage losses). These energy costs can be characterized by the parameters *energy intensity* *EI* and round-trip efficiency $\eta$ (Eq. 2). While the latter is commonly reported and target for improvements (Koohi-Fayegh 2020; Letcher 2016), few studies even report the embodied energy for storage technologies (e.g. Argonne National Lab 2010; Barnhart and Benson 2013; Barnhart et al. 2013). The energy intensity *EI* of storage describes the embodied energy as multiples of storage capacity:

5 $$\begin{aligned} EI &\qquad = \qquad \frac{E_{\rm embodied}}{E_{\rm storage capacity}} \\ E_{\rm embodied} &\qquad \dots\qquad\text{embodied \; energy \; in \; electric \;energy \; equivalents } \\ E_{\rm storage capacity}& \qquad \dots \qquad \text{ nominal \;storage \;capacity} \end{aligned}$$

Knowing the energy “costs” to build storage capacity is, however, fundamental for modelling the transition based on energy balances. In this paper, we estimate the energy intensity, defined as embodied energy per storage capacity (Sect. S4, Eq. 5), for three exemplary storage technologies based on life cycle inventories in literature: Li-ion batteries (LIBs) (Crenna et al. 2021), pumped hydrostorage (PHS) (Wernet et al. 2016) and synthetic methane (syn-CH_4_) (Zhang et al. 2017) (Sect. S4). A comprehensive review for quantifying the energy intensity of other storage technologies is a potential area of future research.

Energy storage technologies are, as all renewable energy technologies, continuously evolving at a high pace. Embodied energy required during manufacture has been declining with technological and organizational improvements. This energy learning rate is established for solar PV (Görig and Breyer 2016; Fraunhofer ISE 2021); however, no data exist for storage technologies.

Even though learning in embodied energy may be significant in the future, there are several reasons, why the projection of historic learning into the future may not be accurate (Grafström et al. 2021). First, this paper is looking into extremely fast transitions, requiring growth in production significantly higher than in the past (Desing and Widmer 2021). Consequently, building production facilities and the mobilization of material resources in sufficient quality and quantity need to be accelerated as well. This increased material flows may even increase energy demand. Furthermore, in fast transitions, there may be not enough time to bring learning effects of increased cumulative production into practice. Second, the embodied energy would reach zero when cumulative production approaches infinity in Eq. 6. It is reasonable to assume that there is a lower limit for embodied energy determined by the thermodynamic energy required to power the necessary physical and chemical processes. It is a potential area for future research to find this limit for embodied energy and a learning curve would need to asymptotically approach this limit. For these reasons, projected historic learning can be seen as optimistic regarding technical progress, whereas a transition without learning can be seen as precautionary. The model (“Transition Model” section) allows to test both cases. The learning rate for energy storage is modelled as follows:

6 $$\begin{aligned} \frac{EI(P_{\rm PV})}{EI_0}= & {} \left( \frac{E_{\rm storage \, capacity} +E_{\rm storage,0}}{E_{\rm storage,0}}\right) ^{\frac{\log (1-LR_{\rm storage})}{\log 2}} \\ E_{\rm storage \, capacity}= & {} P_{\rm demand} \frac{\Delta t_i}{\eta_{\rm out}} = \frac{P_{\rm PV}}{1+\phi \left( \frac{1}{\eta }-1\right) } \frac{\Delta t_i}{\eta_{\rm out}} \end{aligned}$$

Li-ion batteries are the leading storage technology for mobile applications, from smartphones to electric vehicles. Various chemistries exist and are under development, which contain different amounts of Li and other metals, such as Co, Cu, Mn, Ni or Al. Li and Co are currently the main drivers for embodied energy of LIB (Crenna et al. 2021). As Co has a very high energy demand, a battery mix of 1/2 Co-based LIBs (equal mix between NMC111, NMC811 and NCA), which are more suitable for mobile applications, and 1/2 LFP (Lithium-iron-phosphate, Co free), which are more suitable for stationary application, is assumed. While LIBs have a high round-trip efficiency $\eta_{\rm Li-ion} = 0.94$, they have a high energy intensity $EI_{\rm Li-ion}=259$ (i.e. embodied energy is 259 times nominal storage capacity, Sect. S4).

LIBs are currently evolving at a high pace and production capacities are increasing steadily (Chordia et al. 2021; Ziegler and Trancik 2021). However, there is a lack of data regarding the decrease of embodied energy with cumulative production (Chordia et al. 2021). In fact, there is high variability among different studies estimating embodied energy in batteries even with similar production assumptions (Dai et al. 2017; Chordia et al. 2021; Crenna et al. 2021). Chordia et al. (2021) estimate that the production of NMC811 at the scale of 16 GWh/a (assumed representative for 2020) reduces direct operational energy demand of the factory by about 1/2 compared to production of NMC111 cells at the scale of 70 MWh/a (assumed representative for 2012). Materials contribute about 1/2 of overall embodied energy, which is assumed unaffected by cumulative production. Consequently, the energy intensity reduces by 1/4 as cumulative production increases 5-fold between 2012 and 2020.

7 $$\begin{aligned} LR= & {} 1-2^{\frac{\ln \frac{EI_2}{EI_1}}{\ln \frac{E_{\rm cum.prod,2}}{E_{\rm cum.prod,1}}}} \end{aligned}$$

This results in an estimated learning rate for embodied energy of 12 %, assuming that existing production facilities would be able to reduce energy demand as well (optimistic), and 6%, assuming that only newly added manufacturing capacity can implement the reduction in energy demand. In contrast, Dai et al. (2017) find for NMC111 production facility with 2 GWh/a capacity 75% lower energy demand (75 MJ/kWh in electric energy) than (Chorida et al. 2021) for their 16 GWh/a factory. In conclusion, there is not yet enough robust data to estimate embodied energy learning rates for LIB. Ziegler and Trancik (2021) report price learning rate for LIB of 20% for every doubling of cumulative production. Assuming that price learning and embodied energy learning are correlated in a similar way in LIB as in PV, energy learning rate can be estimated. A comparison for PV between learning rate for price (25%) and embodied energy (12.8%) (Fraunhofer ISE 2021) shows that embodied energy learning is about half of price learning. Assuming the same tendency, embodied energy for LIB would decrease by approximately 10 % for doubling cumulative storage capacity, which is in between the estimates based on Chordia et al. (2021) from above. Today’s cumulative LIB production is about $1.1\times 10^{-4}$ TWa in storage capacity (Ziegler and Trancik 2021).

Pumped hydrostorage (PHS) is a well-established technology and is, today, the only mechanical storage employed at large scale. It has a round-trip efficiency of about $\eta_{\rm PHS}=0.76$ (Barnhart and Benson 2013; Koohi-Fayegh 2020; Wernet et al. 2016; Bauer et al. 2007) and energy intensity of $EI_{\rm PHS}\approx 85$ calculated from ecoinvent datasets (Sect. S4). The fraction of embodied energy per output energy can be calculated from ecoinvent datasets (Wernet et al. 2016), which relates to *EI* over the number of charge cycles $n_{\rm cycle}$ and average discharge depth $DD_{\rm average,storage}$:

8 $$\begin{aligned} E_{\rm el,out} \, = \, & {} E_{\rm storage capacity} \cdot DD_{\rm average, storage} \cdot n_{\rm cycle} \end{aligned}$$

9 $$\begin{aligned} EI \, = \, & {} \frac{E_{\rm embodied}}{E_{\rm el,out}}\cdot DD_{\rm average, storage} \cdot n_{\rm cycle} \end{aligned}$$

Current PHS installations have cumulative capacity of about $1.33\times 10^{-4}$ TWa (Department of Energy 2022). As PHS is a mature technology, no learning in embodied energy is assumed.

Suitable sites for traditional pump hydrostorage are not available in all locations and there is increasing opposition to new large-scale hydropower projects. However, technologies are in development that can be installed also on flat land (Heindl 2014, 2021) and may imply a similar energy intensity, as it requires same equipment along with dam construction and tunnelling. Other mechanical energy storage technologies may become available at scale, such as flywheels, compressed air energy storage or gravity storage with solids (Letcher 2016). There is a need to investigate the energy costs for these storage options.

Synthetic methane, produced with hydrogen from electrolysis of water and methanization with carbon from direct air capture (DAC) to $\text{CH}_4$ (Zhang et al. 2017), is taken as the example for chemical storage technologies. It can be easily stored and used in existing infrastructures (e.g. internal combustion engines, gas turbines, domestic heating systems). Like all chemical storage technologies (Alten et al. 2021), synthetic methane has a low round-trip efficiency ($\eta_{\mathrm{CH}_4}=0.22$ (Zhang et al. 2017; Sterner and Specht 2021)) and is therefore energetically expensive to operate. However, it has the lowest energy intensity $EI_{\mathrm{CH}_4}\approx 63$ (Sect. S4), which is calculated from the life cycle inventory provided by Zhang et al. (2017). Other synthetic fuels may have higher efficiency (e.g. $H_2$, which is, however, difficult to store, $\eta_{\mathrm{H}_2} \approx 0.35$ (Pellow et al. 2015; Alten et al. 2021)) or even lower efficiency (e.g. $\text{CH}_3\text{OH}$ if burned in an internal combustion engine $\eta_{\text {CH}_{3}\text {OH}\,\,\text {in\;ICE}} \approx 0.09$ (Alten et al. 2021; Hänggi et al. 2019)).

As for LIB, no data on learning rates for embodied energy of synthetic fuels yet exist. Thus, we estimate the learning rate for embodied energy as half of the learning rate for price (Thema et al. 2019). The learning rate of electrolyzer installation costs had been 10% (Thema et al. 2019); thus for embodied energy, it can be estimated at 5%. Currently installed storage capacity for synthetic fuels is taken as the 2022 projection ($5.8\times 10^{-7}$ TWa) using the exponential growth fitted to historical data by Thema et al. (2019) and a charge time of $\Delta t_{\mathrm{charge,synCH}_{4}}=80{h}$ (Bogdanov et al. 2019).

Other forms of storage, such as thermal storage in phase change materials or electro-magnetic storage such as supercapacitors and superconductors, are still in their development for large-scale applications and not considered here for simplicity.

### Transition Model

The purpose of this model is not to forecast any feasible or implementable transition in great detail, as there are plenty of integrated assessment models trying to capture the real-world complexity of such a transformation. In contrast, we intent to investigate the energy limit for accelerating the energy transition while including energy storage, a limit restricting also real-world transformation pathways. In other words, even when overcoming all social-political challenges, real-world transformations may not surpass this limit. Answering this question allows to radically simplify the problem: looking at global annual average power supply and the energy investments necessary to build solar and storage infrastructure replacing the current fossil energy system. The storage required to level out hourly to seasonal variability depends on demand flexibility, i.e. the willingness and ability to align energy demand patterns with renewable energy supply. We do not investigate to what extent this is possible for specific energy uses, as we are interested in the effect of energy storage demand on the maximum attainable speed of the transition and its effect on cumulative CO_2_ emissions. As such, demand for energy storage and the characteristics of different storage options are the variables in the model.

All other complications are neglected: social, political or economic restrictions, material resource mobilization, geographic (re-)distribution or interdependence with other greenhouse gases, as considering any of them may only slow down but cannot accelerate the transition.

Energy investments are described through the energy payback time, i.e. the time after which a solar installation has provided as much energy to society as was necessary to install it. When accounting for energy storage, the energy payback time for the newly installed renewable energy system needs to be adjusted from $\text {EPBT}_{\rm PV}$ by both the energy intensity of storage and storage losses during the transition. It is determined as follows (see Fig. 1):

10 $$\begin{aligned} P_{\rm PV}= & {} (1-\phi ) P_{\rm demand} + \frac{\phi }{\eta } P_{\rm demand} = P_{\rm demand} \left( 1+\phi \left( \frac{1}{\eta }-1\right) \right) \end{aligned}$$

11 $$\begin{aligned} EPBT&= \frac{E_{\rm embodied}}{P_{\rm demand}} = \frac{P_{\rm PV}\text {EPBT}_{\rm PV} + EI \cdot E_{\rm storage capacity}}{P_{\rm demand}}\\ &= \frac{1}{P_{\rm demand}}\left( P_{\rm demand}\left( 1+\phi \left( \frac{1}{\eta }-1\right) \right) \text {EPBT}_{\rm PV} + EI \cdot P_{\rm demand}\frac{\Delta t_i}{\eta_{\rm out}}\right) \\&= \left( 1+\phi \left( \frac{1}{\eta }-1\right) \right) \text {EPBT}_{\rm PV} + EI\frac{\Delta t_i}{\eta_{\rm out}} \end{aligned}$$

During the transition, only the fraction of replaced fossil energy needs to be stored, which is determined by the fossil replacement factor $\alpha$: $\alpha =0$ describes no fossil replacement, thus also no need to store PV supply during the transition, whereas $\alpha =1$ means full replacement, i.e. the fraction $\phi$ of solar supply needs to go through storage. Therefore, Eq. 11 can be adjusted to reduce the storage losses during the transition:

12 $$\begin{aligned} EPBT= & {} \underbrace{\left( 1+\alpha \phi \left( \frac{1}{\eta }-1\right) \right) }_{\rm storage loss} \text {EPBT}_{\rm PV} + \underbrace{ EI\frac{\Delta t_i}{\eta_{\rm out}} }_{\rm embodied energy of storage} \end{aligned}$$

If, during the transition, fossil replacements are zero ($\alpha =0$), no storage losses would occur during the transition but only afterwards. In this case, the EPBT is enlarged only by embodied energy of storage. In contrast if fossil replacements are $\alpha =1$, storage losses further reduce the available power to grow the solar system and increase EPBT even more.

Embodied energy learning rates for PV had been about 14% per doubling of cumulative PV production between 1974 and 2010 Görig and Breyer (2016) and $12.8{\%}$ between 1996 and 2020 (Fraunhofer ISE 2021). Current PV installations provide 0.07 TW to society (IEA 2020). Consequently, about hundred times more solar panels need to be produced than installed today. Following the learning curve of Fraunhofer ISE (2021), this would allow a reduction in $\text {EPBT}_{\rm PV}$ of about 40% during the transition.

13 $$\begin{aligned} \frac{\text {EPBT}_{\rm PV}(P_{\rm PV})}{\text {EPBT}_{\rm PV,0}}= & {} \left( \frac{P_{\rm PV}+P_{\rm PV,0}}{P_{\rm PV,0}}\right) ^{\frac{\log (1-LR_{\rm PV})}{\log 2}} \end{aligned}$$

To allow for learning rates in the model, the equations from Desing and Widmer (2021) have to be adapted as it was originally built with constant *EPBT*. At the beginning of the simulation ($t=0$), the solar output capacity is zero. One time step $\Delta t$ later, solar panels are installed using fossil power investment $P_{\rm invest}=\beta P_{\rm supply,NR}$, where $\beta$ is the fossil investment factor describing how much of current non-renewable supply $P_{\rm supply,NR}$ is invested in building solar capacity in addition. Utilizing all of currently idle capacity of fossil power plants, this factor is set to $\beta =0.4$ (Desing and Widmer 2021). For simulation time steps thereafter ($t>\Delta t$), the solar engine grows with continued fossil and additional solar investment ($1-\alpha$).

14 $$\begin{aligned} P_{\rm PV}(t=0)= & {} 0 \end{aligned}$$

15 $$\begin{aligned} P_{\rm PV}(\Delta t)= & {} P_{\rm invest} \cdot \frac{\Delta t}{EPBT} \end{aligned}$$

16 $$\begin{aligned} P_{\rm PV}(t>\Delta t)= & {} \underbrace{P_{\rm PV}(t-\Delta t)}_{\mathrm{output capacity at}\;t-\Delta t} \\&+ \underbrace{\left( \underbrace{P_{\rm invest}}_{\rm fossil investment}+\underbrace{P_{\rm PV}(t-\Delta t) (1-\alpha )}_{\rm solar investment}\right) \cdot \frac{\Delta t}{EPBT(P_{\rm PV}(t-\Delta t))}}_{\mathrm{additions at}\;t} \end{aligned}$$

The transition is completed, when the solar engine is big enough to supply the demand by society. The required size of the solar system depends on storage technology and stored fraction, as it has to provide the storage losses in addition to demand by society (see Fig. 1).

17 $$\begin{aligned} P_{\rm PV,required}= P_{\rm demand}+ P_{\rm storage\; loss} \\ = P_{\rm demand} \left( 1+\phi \left( \frac{1}{\eta }-1\right) \right) \end{aligned}$$

## Results

**Fig. 3:**
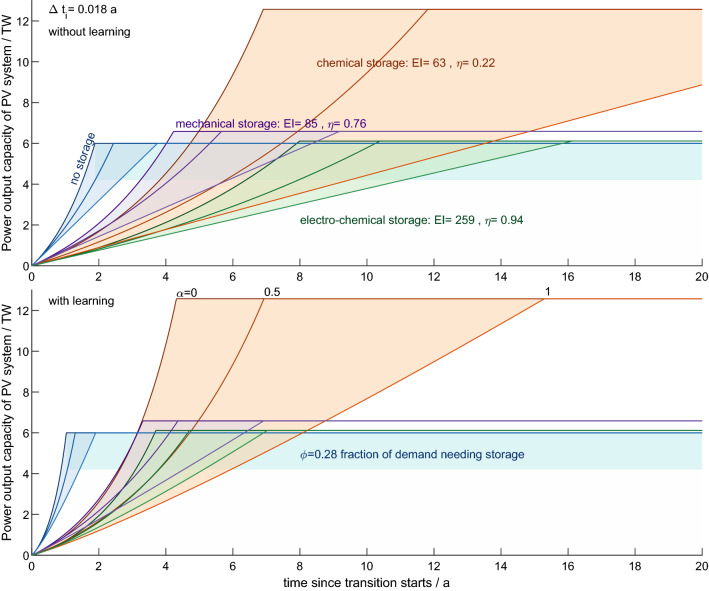
Transition dynamic for $\phi =0.28$ and $\Delta t_i = 0.018\,\text{a}$ (which is approximately one week), which corresponds to the storage demand as modelled in Bogdanov et al. (2019). Transition without storage (Desing and Widmer 2021) in blue, chemical storage (orange), mechanical storage (violet) and electro-chemical storage (green). Fossil replacement factor $\alpha =0$ allows an exponential and therefore fastest growth, whereas $\alpha =1$ leads to a linear growth. The upper panel shows the transition dynamic without learning, and the lower panel shows with learning (Color figure online)

Adding energy storage to fast and complete energy transition pathways (Desing and Widmer 2021) has two main effects on the energy system (Fig. 3): (I) additional energy for building storage capacity has to be provided, necessarily slowing down the building of solar capacity. And (II) the solar power has to provide for storage losses, therefore increasing the required solar capacity. Both requirements are slowing down the transition and increase cumulative fossil carbon emissions. A low fossil replacement factor $\alpha$ allows for an exponential growth of the solar engine (Fig. 3), while full fossil replacement ($\alpha =1$) slows the transition down to linear growth, as only constant fossil investments are available to build the solar engine. Storage has to be operated during the transition too, which further reduces the energy output of the solar supply system.

**Fig. 4:**
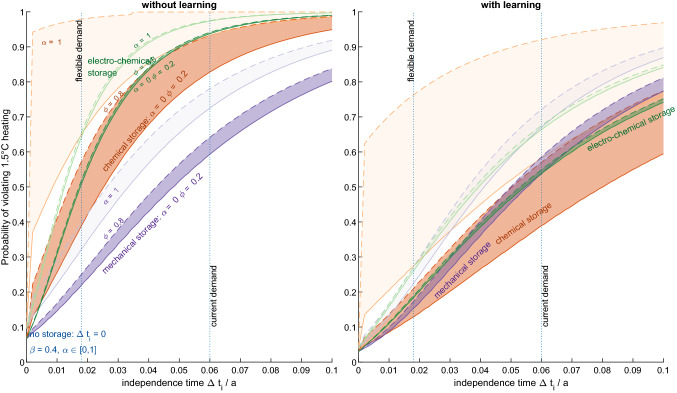
Probability of violating 1.5 °C as a function of independence time $\Delta t_i$. Left panel shows the results for transitions without learning (precautionary) and the right panel shows with learning (optimistic). Blue line indicates the probabilities and transition times for a transition without storage (Desing and Widmer 2021). Probability of violating 1.5 °C increases with $\phi$, $\Delta t_i$ and $\alpha$, however, depending on storage technology groups differently: mechanical storage (violet), chemical storage (orange) and electro-chemical storage (green). “Current demand” dotted line denotes a scenario where energy demand profiles remain as today (Pleßmann et al. 2014), whereas “flexible demand” dotted line denotes the scenario of Bogdanov et al. (2019) including demand flexibility (Color figure online)

Larger storage capacities—described by independence time $\Delta t_i$—slow down the growth of the solar engine, whereas increasing storage throughput ($\phi$) necessitates to build the solar engine larger in order to compensate for increased storage losses. The former effect is most pronounced for storage technologies with high energy intensity (e.g. electro-chemical storage), while the latter is most influential for storage technologies with low round-trip efficiency (e.g. chemical storage). Both increase the probability to exceed 1.5 °C heating (Fig. 4).

The fastest possible transition without storage and without learning achieves a minimally possible probability to exceed 1.5 °C of $P_{\rm v} = 0.07.$ At the same cumulative CO_2_ emissions, the probability is substantially lower than previously determined [20% (Desing and Widmer 2021)]. The difference roots in the update of the probability distribution provided by the latest IPCC Sixth Assessment Report. Due to a narrower distribution, the same cumulative emissions now show a lower probability to exceed 1.5 °C (Fig. S1). At the same time, delaying climate action increases the probability to exceed 1.5 °C much more rapidly. When including learning for embodied energy of PV, the transition can be achieved even faster, reducing the lowest possible probability to exceed 1.5 °C to $P_{\rm v} = 0.03.$

Increasing storage demand raises the probability to exceed 1.5 °C substantially above a transition without storage (Fig. 4). Energy intensity of storage is most influential on the speed of the transition. Even though chemical storage has a low round-trip efficiency, it comes at low embodied energy, enabling a fast transition when fossil replacement $\alpha$ is small or zero. At large $\alpha$, in contrast, the transition with chemical storage is significantly slowed down due to the need to provide for storage losses during the transition. Increasing storage fraction $\phi$ again increases storage losses and thus cumulative emissions. In contrast, electro-chemical storage technologies typically have high round-trip efficiencies, but also a high energy intensity. Increasing the storage fraction has a much smaller effect than increasing storage capacity.^1^ Mechanical storage allows fastest transitions due to the low energy intensity and comparatively high round-trip efficiency in the case without learning. When learning is considered, all three exemplary storage options perform similarly for $\alpha =0,$ while for $\alpha =1$ chemical storage performs worst. When aiming at a low probability to exceed 1.5 °C (i.e. apply the precautionary principle), storage demand needs to be minimized ($\Delta t_i<0.01\,\text{a}$, $\phi <0.2$), the transition starts as soon as possible and no fossil replacement during the transition ($\alpha =0$).

For fulfilling current electric energy demand profiles, independence time $\Delta t_i=0.06\,\text{a}$ and storage fraction $\phi =0.5$ are necessary [“current demand” in Fig. S3 and Table S1 based on Pleßmann et al. (2014)]. This scenario with fixed demand leads to a probability to exceed 1.5 °C between 60 and 100% without learning and 50% and 80% with learning. When allowing demand flexibility and sector coupling, storage demand reduces to independence time $\Delta t_i=0.018\,\text{a}$ and storage fraction $\phi =0.28$ [“flexible demand” in Fig. S3 and Table S2 based on Bogdanov et al. (2019)]. The probability to exceed 1.5 °C decreases to the range between 22 and 76% without learning and 14 to 36% with learning. Consequently, satisfying “current” and “flexible” demand for storage significantly increases the probability to exceed 1.5 °C over a transition without storage.

## Discussion

Increasing storage capacity and energy throughput increases the probability to violate 1.5 °C heating (Fig. 4). PHS (as a representative technology for mechanical storage) increases climate risks the least when disregarding learning, as it has both a relatively low energy intensity and high round-trip efficiency. However, topographically suitable sites are rare and new technologies independent of topography are still in their infancy, which makes PHS difficult to scale. LIBs are a good option for very small independence times of about 1 day or two ($\Delta t_i < 0.005$), whereas synthetic methane is feasible as long-term, seasonal storage with low throughput. While this general tendency is regularly confirmed in literature, it is important to note that each of these storage technologies have a substantial effect on cumulative CO_2_ emissions and consequently the probability to exceed 1.5 °C (Fig. 4)—regardless of learning taking place or not and already at very short independence times. Energy investments are commonly not considered in energy transition models (Capellán-Pérez et al. 2020), yet our findings show that energetic costs of storage, both embodied and operational, are important to consider when aiming for fast transition pathways. Implementing storage to satisfy current demand patterns will even at the fastest possible transition lead to more than 50% chance of exceeding 1.5 °C heating. The results show that minimizing storage demand is imperative for climate protection.

Research and development for novel storage technologies need to focus on reducing energy intensity while increasing round-trip efficiency. However, time is pressing, as every year of climate inaction increases cumulative emissions by about 42 Gt (Friedlingstein et al. 2019; Le Quéré et al. 2020). For example, delaying the start of the transition by another 5 years (2028) increases the minimally attainable probability to exceed 1.5 °C heating to 40%. Consequently, the transition needs to commence immediately with readily available and scalable technologies. New storage technologies can, once market ready, allow to increase storage capacity and throughput in the future.

**Fig. 5:**
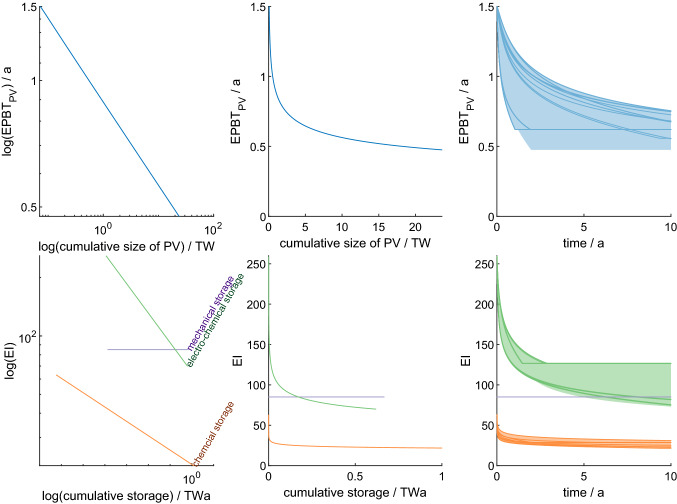
Reduction of embodied energy demand according to the projection of historic learning rates for PV (upper row) and energy storage (lower row; green for electro-chemical, orange for chemical and violet for mechanical storage). Left column shows the embodied energy in dependence of cumulative installations in a log–log grid, while the middle column shows the same data in a linear grid. The right column shows the range of results for embodied energy decline as a function of time in all different simulation runs

Learning can have a profound effect on accelerating the energy transition (Figs. 3 and 4). When including learning, transition times can be approximately halved compared to the situation without learning. However, substantial reduction of embodied energy for both PV and storage technologies has to occur within a very short time (right column in Fig. 5)—for PV up to 2/3 within less than 2 years. Similarly, energy intensity of batteries and synfuels has to reduce significantly within the first few years of the transition. It is questionable that such rapid learning will occur in practice. Not only would large manufacturing capacities be added with monthly improved technologies, but also all prior production facilities needed to be updated monthly. Learning has undoubtedly large potential to reduce the probability to exceed 1.5 °C heating by improving technologies; however, historic learning rates projected to the context of fastest possible transitions have to be considered optimistic (Grafström et al. 2021). It remains to be demonstrated what level of learning is feasible in the very short time frame of a fast transition. Such feasible learning rates are expected to be within the “precautionary” scenario without learning and the “optimistic” scenario with projected historic learning.

Apart from improving technology, the second possibility to reduce climate risks is to minimize the demand for storage, both in terms of independence time and fraction of demand needing storage. This, however, requires a fundamental re-thinking of the way we use energy in society. It includes currently discussed demand flexibility options through smart grids, sector coupling and grid distribution (Bogdanov et al. 2021). Beyond that, storage demand can be further reduced by changing the current demand-driven to a supply-driven energy system, where demand is more synchronized with solar supply. The consequences for society of such a paradigm shift are discussed in “Conclusions and Implications” section.

As the model aims at finding the energy limit for accelerating the energy transition when including storage, it necessarily neglects various relevant aspects: storage demand can be reduced to some extent by transporting energy via electric energy grids (Brown et al. 2018). Efficient long-distance energy transmission is rapidly expanding (Joint Research Center 2020) and will gain importance to minimize storage needs by levelling regional, e.g. due to local cloud cover, or in the future even intercontinental solar irradiation differences, i.e. day and night (West to East) and winter and summer (North to South). The energy requirements and implications on the transition remain to be investigated.

The balancing effect of the existing renewable energy system, most notably dispatchable hydropower and biomass, and flexibility potentials when integrating energy sectors (Bogdanov et al. 2021) are neglected. It, however, cannot make storage obsolete entirely; thus, it is still relevant to determine the influence of storage on the transition. A more detailed analysis is necessary when determining the achievable minimum for storage demand.

A gradual decarbonization (i.e. $\alpha >0$) allows to build and operate storage much later, as in the beginning fossil capacity can still be used to balance variable solar supply (Victoria et al. 2019). Yet, in very fast transitions, this influence is considered insignificant given the short time for building the necessary renewable infrastructure. Further research is necessary to investigate other physical and societal limits to the attainable speed of the transition, such as the mobilization of the necessary material and human resources such as knowledge and skills.

The results of this study show the significance to include energy investments when modelling energy transitions, all the more in view of the rapidly exhausting carbon budget. There is a need to enhance existing literature by adding research on energy costs of current and future energy storage and other key technologies and include them into integrated assessment modelling (Capellán-Pérez et al. 2020).

## Conclusions and Implications

**Fig. 6:**
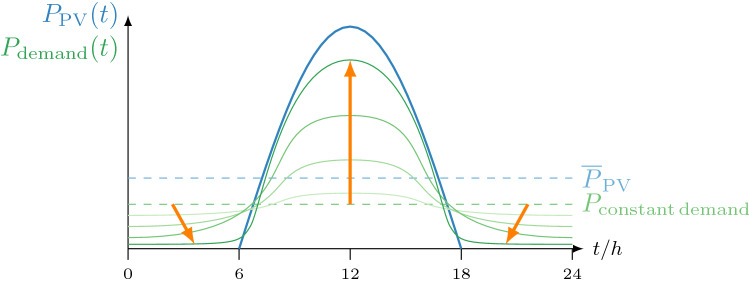
Sunflower society aligns (orange arrows) the demand (green lines) with renewable supply (blue line) throughout the course of a day (Color figure online)

Exploring energy constrained transition pathways can help to identify what is still possible if society could manage to overcome all material, social and economic constraints for safeguarding its own future. At the same time, it also identifies what lies beyond reach, i.e. the transition cannot be accelerated beyond the energy limit. As shown in this study, adding energy storage significantly reduces the attainable speed of the energy transition due to its high energy costs. Consequently, increasing storage demand reduces the probability to stay below 1.5 °C heating. Safeguarding a hospitable climate for future generations with high confidence thus requires to minimize the demand for storage much below of what would be necessary to meet current demand patterns. This can be achieved by combining supply side measures, such as sector coupling and smart grids, with aligning demand patterns to solar supply (Fig. 6). Since a substantial share of the fossil energy uses considered hard-to-avoid, such as aviation mainly benefit a minority of the world’s population (Oswald et al. 2020), the energy transition will also need to reshape the energy demand including its allocation, timing and distribution. Any delay in climate action and any real-world constraint reducing the attainable speed of the transition will make reducing and aligning demand with solar supply more relevant.

Following the course of the sun, just like sunflowers do, society can schedule its most energy-intensive activities around midday and summer, while reducing the demand at night and in winter to the bare minimum. This had been the rule throughout life’s and most of humanity’s history. For instance, plants are most active during peak sun hours and in summer, while dormant in the night and during winter. And consequently, agricultural societies had been following the activity cycles of plants. An industrial society can, in principle, also align its energy demand to solar supply.

Implementing a sunflower society requires a paradigm shift in society towards supply-driven energy systems. The following will outline founding principles for such a change, termed here the “sunflower society”.

Avoided energy demand also avoids storing it. Reducing energy demand (Millward-Hopkins et al. 2020; Grubler et al. 2018) is especially important for energy uses that cannot be shifted to sun hours, e.g. lighting and emergency hospitals. Here, it is most important to apply and evolve as-efficient-as-possible technologies and use it only when absolutely necessary. Reducing lighting has the additional benefit of avoiding light pollution obstructing wildlife. In addition, it is also particularly important for any energy-intensive process. For example, reducing the demand for aluminium and shifting from primary production to increased recycling has the potential to significantly reduce both energy demand and the pressure on limited resources (Desing et al. 2020).

The need for daily storage can be reduced by concentrating economic activity and therefore energy demand around peak sun hours across all demand areas. Technologies can support and enable the alignment of demand with supply. For example, through the shift away from continuously operating technologies—like steel making in blast furnaces—to batch operations during peak sun hours—like it will be possible for hydrogen steel making in electric arc furnaces (Ahman et al. 2018). Even processes that are designed for continuous operations, like blast furnaces, can—in principle—use their thermal inertia to reduce energy demand throughout the night. This alignment can be stimulated through hourly energy tariffs, which are lower during peak sun hours, simply inverting today’s practice of providing incentives for using energy during the night.

It further means to change behavioural patterns—like reducing mobility demand and shifting to grid-connected modes of transport. Charging battery electric vehicles during the night would require double storage: one for storing solar energy harvested during the day and one in the car, which is charged in the night. Grid-connected modes of transport, like trolleybuses and trains, can provide the same transport service during the day without storage.

A sunflower society would also align its demand with seasons in higher latitudes: concentrating energy-intensive activities during summer, while reducing energy demand in winter. Space heating, for example, is necessary during the coldest months, when irradiation is lowest. Heating demand can be reduced by shrinking the floor space per inhabitant or building multi-family houses, which have a smaller surface-to-volume ratio. Much of the demand for storage beyond daily variation shall ideally be avoided completely. This can, for example, be achieved through shifting from active energy demand, e.g. residential heating or online media, to passive energy demand embodied in materials, e.g. insulation materials or printed media. The production and recycling of these materials can be scheduled in summer, effectively acting as energy storage.

Over-sizing solar capacity can contribute to avoiding seasonal storage. As the energy intensity of solar PV systems is much lower than for energy storage, it is energetically cheaper to build. PV systems can be oversized in order to provide the daily demand also on the day of the year with lowest irradiation. During the rest of the year, excess power can be either curtailed or used to power the next big human endeavour after the energy transition: cleaning up the atmosphere (Desing et al. Submitted). Excess CO_2_ in the atmosphere can be removed with direct air capture and carbon storage technologies (Gambhir and Tavoni 2019; Fasihi et al. 2019; Breyer et al. 2019; Deutz and Bardow 2021) and stored permanently, thus reverting anthropogenic climate heating and stabilizing the climate in the long run.

## Supplementary Information

Below is the link to the electronic supplementary material.

Supplementary file 1 (pdf 1171 KB)

## Data Availability

Calculation code is available on zenodo: https://doi.org/10.5281/zenodo.5524262

## Abbreviations

DD (1): Average discharge depth of storage cycles
$E_{\rm embodied}$ (J): Energy necessary to build infrastructure, i.e. embodied in infrastructure
$E_{\rm el,out}$ (J): Electric energy output from storage over the whole lifetime
$E_{\rm PV,embodied}$ (J): Energy necessary to build PV infrastructure, i.e. embodied in PV infrastructure
$E_{\rm storage\;embodied}$ (J): Energy necessary to build storage infrastructure, i.e. embodied in storage infrastructure
$E_{\rm storage\;capacity}$ (J): Energy storage capacity, i.e. energy that can be stored in storage infrastructure
EI (1): Energy intensity of storage, i.e. embodied energy in multiples of storage capacity
EPBT (a): Energy payback time, i.e. time after which as much energy had been supplied to society as was necessary to build the infrastructure
$\text {EPBT}_{\rm PV}$ (a): Energy payback time for PV alone
LR (1): Learning rate for embodied energy
$n_{\rm cycle}$ (1): Cycle life of storage technology
$P_{\rm PV}$ (W): Annual average power harvested by PV system
$P_{\rm demand}$ (W): Annual average power demand by society
$P_{\rm invest}$ (W): Annual average fossil power invested to build renewable infrastructure during the transition.
$P_{\rm in}$ (W): Annual average power input to storage
$P_{\rm out}$ (W): Annual average power output from storage
$P_{\rm storage,loss}$ (W): Annual average power lost for storage
$P_{\rm stored}$ (W): Annual average power stored in energy storage
$P_{\rm direct}$ (W): Annual average power provided to society directly
$\alpha$ (1): Fossil replacement factor
$\beta$ (1): Fossil investment factor
$\phi$ (1): Fraction of power demand $P_{\rm demand}$ provided through storage
$\phi$ (1): Fraction of PV power going to storage
$\eta$ (1): Round-trip efficiency of energy storage
$\eta_{\rm in}$ (1): Input efficiency of energy storage, i.e. energy stored over energy fed to storage (“charging efficiency”)
$\eta_{\rm out}$ (1): Output efficiency of energy storage, i.e. energy provided from storage over energy stored (“discharging efficiency”)
$\Delta \;t$ (a): Time step of simulation
$\Delta \;t_i$ (a): Independence time, i.e. time during which demand can be supplied through storage alone.
$\Delta \;t_{\rm charge}$ (a): Minimal charging/discharging time, i.e. time after which the whole storage capacity can be discharged at maximum power.

## Footnotes

1 Note, the effect of changing storage fraction and storage capacity on the lifetime, and therefore replacement needs, is not considered as for most fast transitions, replacements will not be necessary during the transition. For modelling the behaviour of the system after the transition, EoL replacements need to be considered, which is a potential area for further research.
