# Supplementary material for: How Much Energy Storage can We Afford? On the Need for a Sunflower Society, Aligning Demand with Renewable Supply
Source: Biophys Econ Sust. 2022 Apr 28;7(2):3. doi: 10.1007/s41247-022-00097-y (PMC9113673; doi:10.1007/s41247-022-00097-y)
Supplement: Supplementary file 1 — (pdf 1171 KB) [file 41247_2022_97_MOESM1_ESM.pdf]

Supplementary information to “How much energy storage can we afford? On the need for a sunflower society, aligning demand with renewable supply”

Harald Desing*[1], Rolf Widmer[1]

[1] Empa - Swiss Federal Laboratories for Material Science and Technology, Lerchenfeldstrasse 5, 9014 St. Gallen, Switzerland

* corresponding author: harald.desing@empa.ch

Calculation code for LCA of energy storage options (in brightway2) as well as for the energy transition model including storage (in Matlab) can be found here: https://doi.org/10.5281/zenodo.5524262.

S1 Additional figures

In Fig. S1 log-normal probability distributions fitted to the remaining carbon budget data in IPCC Sixth Assessment Report (AR6) (IPCC 2021) and Special Report on 1.5 °C warming (SR1.5) (IPCC 2018) is compared. Log-normal distributions fit very well to the provided data points ($R^2 > 0.98$). It becomes apparent, that the uncertainty had been reduced in AR6 compared to SR1.5. This is the reason why the fastest possible transition without storage can achieve an even lower probability of violating 1.5 °C target than in our previous study (Desing and Widmer 2021).

Fig. S2 shows results for the probability of violating 1.5 °C target for the different considered storage technologies, with and without learning and in dependence of storage demand. The 3D surfaces show the dependence and tendencies of the probability to exceed 1.5 °C from independence time Δt_i, storage fraction ϕ and fossil replacement factor α.

Fig. S3 shows the same results as in Fig. 4 for the probability distribution of 1.5 °C heating as provided by the Special Report on 1.5 °C (IPCC 2018). Note, that the probability to exceed 1.5 °C in the case without learning and no storage corresponds to Desing and Widmer 2021, however, as the transition starts one year later (2023 instead 2022), it is slightly higher. The difference in the two figure comes from the update in the probability distribution describing the remaining carbon budget for staying below 1.5 °C (Fig. S1).

S2 Demand profile estimation

To estimate the maximum fraction of annual average daily harvest that needs to be stored, we approximate the irradiation profile over the course of a day as a cosinus function:

$$P_{\mathrm{PV}}(\psi) = \begin{cases} -P_{\mathrm{PV,max}} \cos\psi & \text{if} \quad -\cos\psi \leq 0 \\ 0 & \text{else} \end{cases} \tag{18}$$

where $\psi = 0$ corresponds to midnight (0 a.m.), $\psi = \pi$ to midday (12a.m., see figure S4).

The average solar power over 24 h is:

$$\overline{P}_{\mathrm{PV}} = \int_{\frac{\pi}{2}}^{\frac{3\pi}{2}} P_{\mathrm{PV}}(\psi) d\psi = \frac{P_{\mathrm{PV,max}}}{\pi} \tag{19}$$

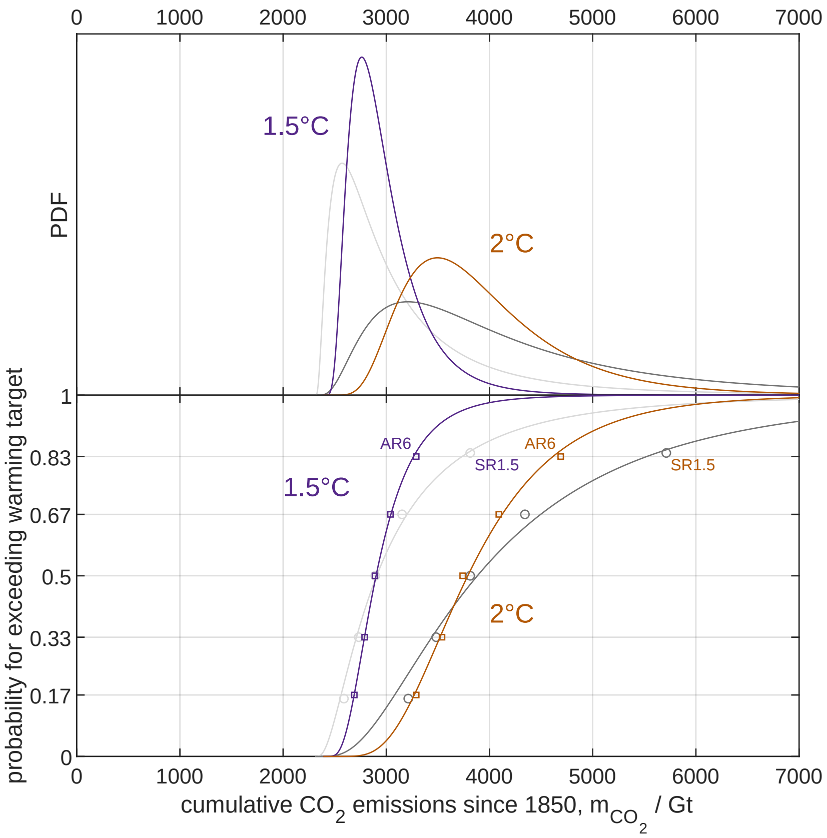

Figure S1: Comparison of the probability density function (upper panel) and cumulative probability distribution (lower panel) of log-normal distributions fitted to the remaining carbon budget data in IPCC Sixth Assessment Report (AR6) (IPCC 2021) and Special Report on 1.5 °C warming (SR1.5) (IPCC 2018).

If we assume the demand to be constant over the day, any power in excess of the average power output would need to be stored.

$$E_{\text{daily storage,max}} = \int_{\psi_1}^{\psi_2} P_{\text{PV}}(\psi)d\psi - \overline{P}_{\text{PV}}(\psi_2 - \psi_1) \tag{20}$$

$$E_{\text{PV,daily}} = \int_{\frac{\pi}{2}}^{\frac{3\pi}{2}} P_{\text{PV}}(\psi)d\psi \tag{21}$$

$$\phi_{\text{daily}} = \frac{E_{\text{daily storage,max}}}{E_{\text{PV,daily}}} = 0.587 \tag{22}$$

The independence time to store daily variations at constant demand (Fig. S4) is

$$\Delta t_i = 1 - \frac{\psi_2 - \psi_1}{2\pi} = 14.5\,\text{h} \tag{23}$$

Demand profiles of current electricity demand across the globe show a slightly higher demand during the day than during the night (Fig. 2). Therefore the fraction of daily demand stored is smaller. Analysing data for electricity demand in 2015 (Brinkerink et al. 2021)[2] yields $\phi_{\text{daily,global}} = 0.54$ for the global average of daily demand needing storage (see Matlab code; data from (Brinkerink et al. 2021) needs to be

[2]Data can be accessed here: https://dataverse.harvard.edu/file.xhtml?persistentId=doi:10.7910/DVN/CBYXBY/HXZGSB&version=6.2

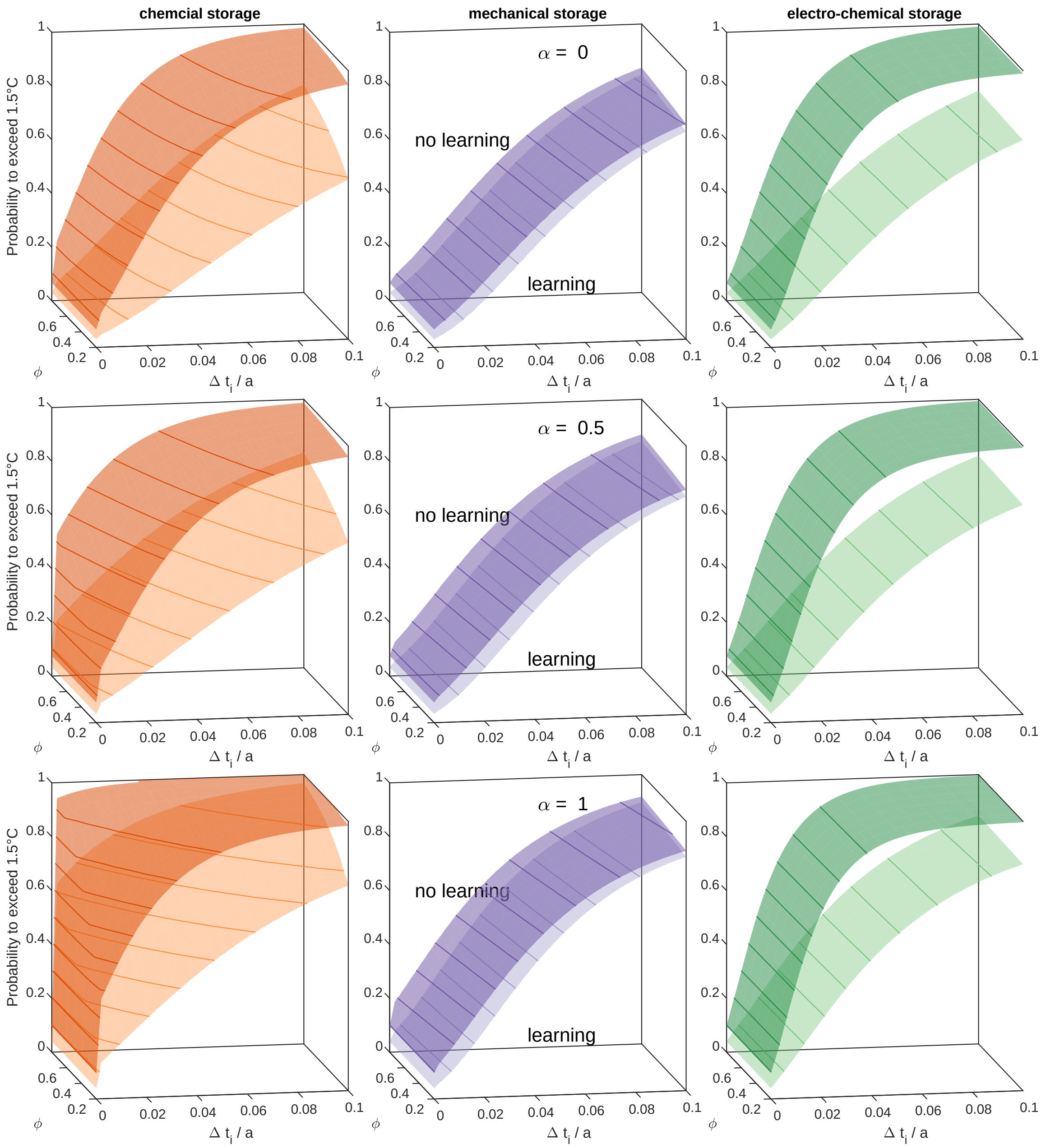

Figure S2: Probability of violating 1.5 °C target in dependence of independence time Δt_i and storage fraction ϕ. The right column shows results for chemical, the middle column for mechanical and the left column for electro-chemical storage. The upper row shows results for $\alpha = 0$, the middle row for $\alpha = 0.5$ and the bottom row for $\alpha = 1$. The darker, upper surface represents the results for scenario without learning and the lighter lower surface for with learning.

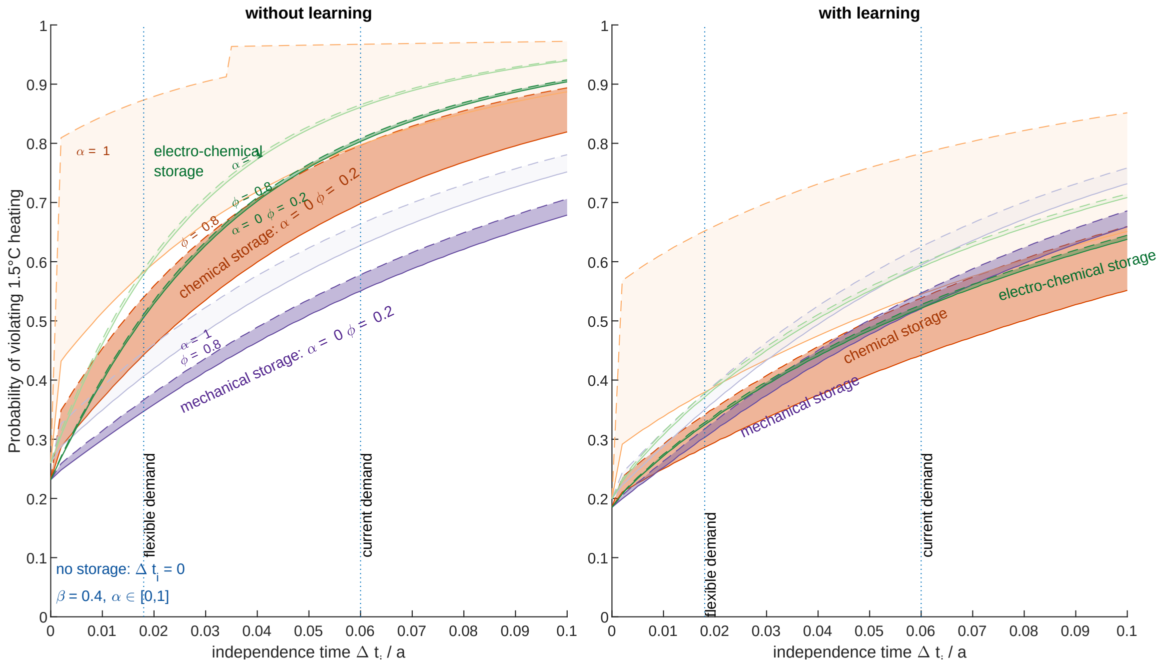

Figure S3: Probability of violating 1.5 °C as a function of independence time Δt_i according to Special Report 1.5 °C (IPCC 2018). Left panel shows the results for transitions without learning (precautionary) and the right panel shows with learning (optimistic). Blue line indicates the probabilities and transition times for a transition without storage (Desing and Widmer 2021). Probability of violating 1.5 °C increase with ϕ, Δt_i and α, however, depending on storage technology groups differently: mechanical storage (violet), chemical storage (orange) and electro-chemical storage (green). "Current demand" dotted line denotes a scenario where energy demand profiles remain as today (Pleßmann et al. 2014), whereas "flexible demand" dotted line denotes the scenario of Bogdanov et al. 2019 including demand flexibility. Results for cumulative CO_2 emissions are identical to Fig. 4.

shifted by time zones to local time, which is assumed to be approximately solar time). Mozambique has the demand profile, which is most aligned to solar supply, thus requiring the lowest $\phi_{\text{daily,MOZ}} = 0.45$. In contrast, the southern region of Russia has the highest demand for storage with $\phi_{\text{daily,RUS-SO}} = 0.59$. This is even higher than for the constant demand (Equation 22), as here demand is lower during the day and higher in the night. Note, that these demand profiles only concern electricity and do not reflect demand for mobility or fuels.

For mobility, the storage fraction is estimated through the demand profile reported in Brown et al. 2018b. Matching the normalized mobility demand profile to normalized solar supply, a fraction of $\phi = 0.39$ needs to be stored (Fig. S5).

S3 Storage demand estimates from literature

Pleßmann et al. 2014 provide estimates for storage capacity and throughput when global electricity would fully decarbonise and be replaced with wind and solar. The scenario is cost optimised and is based on historic electricity demand profiles. Bogdanov et al. 2019 present a scenario for electrifying and decarbonising all energy uses globally and present data for storage demand in a cost optimal way. This scenario allows demand flexibility and sector coupling to reduce the need for storage. For both scenarios

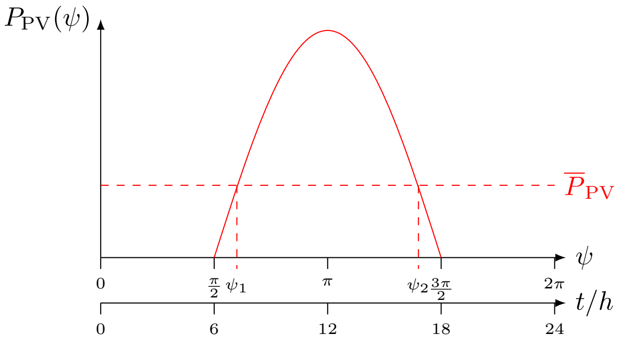

Figure S4: For the approximation of maximum daily storage demand: yearly average solar power profile (solid line) matching constant demand ($\overline{P}_{\mathrm{PV}}$, dashed line).

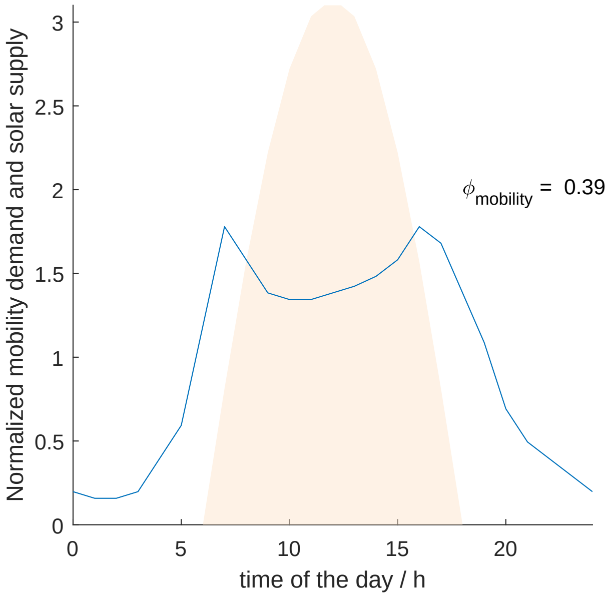

Figure S5: Normalized mobility demand pattern (Brown et al. 2018b) in comparison to normalized solar supply (orange surface).

the storage fraction ϕ and independence time Δt_i have to be calculated using the following approach:

$$P_{\text{in}} = \frac{P_{\text{out}}}{\eta} \tag{24}$$

$$P_{\text{stored}} = \frac{P_{\text{out}}}{\eta_{\text{out}}} \tag{25}$$

$$P_{\text{storage,loss}} = P_{\text{in}}(1-\eta) \tag{26}$$

$$P_{\text{demand}} = P_{\text{supply}} - P_{\text{storage,loss}} \tag{27}$$

$$\phi = \frac{P_{\text{out}}}{P_{\text{demand}}} \tag{28}$$

$$\Delta t_i = \frac{E_{\text{storage capacity}}\eta_{\text{out}}}{P_{\text{demand}}} \tag{29}$$

The values are presented in Tab. S1 and S2.

Current electricity demand			syn-CH_4	battery	thermal	total
storage capacity	$E_{\text{storage capacity}}$	TWh	1690	1.5	73.6	1765.1
storage output	P_{out}	PWh/a	1.96	0.42	4.80	7.18
input efficiency	η_{in}	-	0.41	0.97	0.87	0.702
output efficiency	η_{out}	-	0.60	0.97	0.42	0.475
efficiency	η	-	0.246	0.941	0.365	0.333
storage input	P_{in}	PWh/a	7.97	0.45	13.14	21.55
stored energy	P_{stored}	PWh/a	3.27	0.43	11.43	15.13
storage losses	$P_{\text{storageloss}}$	PWh/a	6.01	0.03	8.34	14.37
power supply	P_{supply}	PWh/a				28.60
power demand	P_{demand}	PWh/a				14.23
fraction of power stored	ϕ	-				0.5
independence time	Δt_i	a				0.06

Table S1: Data and results for calculating storage fraction ϕ and independence time Δt_i from the scenario of Pleßmann et al. 2014 with current electricity demand profiles and considering electricity only.

Flexible demand and sector coupling			syn-CH_4	battery	thermal	total
storage capacity	$E_{\text{storage capacity}}$	TWh	1000	48	3	1051
storage output	$P_{\text{storage,out}}$	PWh/a	0.27	14.4	0.25	14.9
input efficiency	η_{in}	-	0.41	0.97	0.87	0.931
output efficiency	η_{out}	-	0.60	0.97	0.42	0.939
efficiency	η	-	0.25	0.94	0.37	0.874
storage input	$P_{\text{storage,in}}$	PWh/a	1.08	15.29	0.70	17.07
stored energy	P_{stored}	PWh/a	0.44	14.84	0.60	15.88
storage losses	$P_{\text{storageloss}}$	PWh/a	0.81	0.90	0.44	2.16
power supply	P_{supply}	PWh/a				56
power demand	P_{demand}	PWh/a				53.8
fraction of power stored	ϕ	-				0.28
independence time	Δt_i	a				0.018

Table S2: Data and results for calculating storage fraction ϕ and independence time Δt_i from the scenario of Bogdanov et al. 2019 with flexible energy demand and sector coupling.

S4 Energy costs for storage

In this paper, we analyse three main sets of storage technologies: LIB, pumped hydro storage and synthetic fuels. Tab. S3 summarizes the energy performance of the considered storage systems.

S4.1 Li-ion batteries

Round-trip efficiency is estimated at $\eta_{\text{Li-ion}} = 0.94$ for all four different cell chemistry types (i. e. NMC111, NMC811, NCA and LFP) (Crenna et al. 2021; Koohi-Fayegh and Rosen 2020). Storage losses are assumed to be equal at input and output, as most of the losses can be traced back to Ohm's resistance.

Energy intensity is calculated using the life cycle inventories from Crenna et al. (Crenna et al. 2021) for the three cell chemistries NMC111, NMC811 and NCA and ecoinvent v.3.8 (Wernet et al. 2016) as a background database. The calculations for LFP is based on preliminary data (Crenna 2022) and ecoinvent v.3.8. Embodied energy is calculated as the cumulative energy demand in electric energy equivalents (i. e. all contribution to CED converted into electric energy with state-of-the-art technologies, see (Desing et al. 2020)) per unit of storage capacity. Calculation is performed in brightway2 (Mutel 2017) and python code can be found online. Energy demand for Cobalt production is a main driver for total energy demand in NMC111, NMC811 and NCA. The average values are calculated as the average of the three Co-based batteries (NMC111, NMC811 and NCA) and the Co-free LFP.

S4.2 Pumped hydro storage

Round-trip efficiency is taken from ecoinvent v.2, which is based on a review of Swiss hydro power plants, including pumped hydro installations (Bauer et al. 2007). New installations may have round-trip efficiencies up to 85 % (Barnhart and Benson 2013; Grote and Feldhusen 2005; Koohi-Fayegh and Rosen 2020). Turbine efficiency (output) is usually higher than pumping efficiency (input), therefore we assume $\eta_{\text{output}} = 0.9$ and $\eta_{\text{input}} = 0.84$

The energy intensity is calculated with Equation 9 from embodied energy over energy stored over its lifetime, which can be calculated from ecoinvent (Bauer et al. 2007; Wernet et al. 2016). Calculation code in brightway2 can be found online. Depending on the assumption regarding lifetime (70 a or 80 a at daily cycles), the energy intensity is slightly different and the average is taken for the calculations.

S4.3 Synthetic methane

Efficiencies are taken from Zhang et al. 2017, who analyse syn-CH_4 production using PEM electrolyser, CO_2 from direct air capture and combusted in a conventional gas turbine. Alternatively, syn-CH_4 could also be combusted in a combined cycle power plant with higher efficiency (Sterner and Specht 2021). The average is used for the further calculations. The energy intensity is calculated from the life cycle inventory modelling of Zhang et al. 2017. Calculation code in brightway2 can be found online.

storage technology	round-trip efficiency	input efficiency	output efficiency	cycle life	depth of discharge	energy intensity (2021)	learning rate for energy intensity	installations (2021)	storage capacity / nominal storage power (Bogdanov et al. 2019)	Sources
	η	η_{in}	η_{out}	n_{cycle}	DD	EI_0	$LR_{storage}$	$E_{storage,0}$/TWh	$\Delta t_{charge}/h$	
Li-Ion battery NMC111	0.94	0.97	0.97			457				Crenna et al. 2021; Wernet et al. 2016
Li-Ion battery NMC811	0.94	0.97	0.97			323				Crenna et al. 2021; Wernet et al. 2016
Li-Ion battery NCA	0.94	0.97	0.97			339				Crenna et al. 2021; Wernet et al. 2016
Li-Ion battery LFP	0.94	0.97	0.97			145				preliminary data
**Li-Ion battery average**	0.94	0.97	0.97			259	0.10	0.99	6	Ziegler and Trancik 2021
pumped hydro storage (PHS)	0.76	0.84	0.90	25000	0.5	81				ecoinvent 3.7.1
PHS, increased lifetime	0.76	0.84	0.90	29200	0.5	90				ecoinvent 3.7.1
**PHS average**	0.76	0.84	0.90			85	0	1.17	8	Department of Energy 2022
synthetic CH4 (PEM + DAC)	0.16	0.41	0.40	1000	1	63				Wernet et al. 2016; Zhang et al. 2017
synthetic CH4 (PEM + DAC + combined cycle)	0.27	0.45	0.60							Sterner and Specht 2021
**synthetic CH4 average**	0.22	0.43	0.50			63	0.05	0.005	80	Thema et al. 2019

Table S3: Energy performance of selected storage technologies. Background database for LCA calculations is ecoinvent v.3.7.1 (Wernet et al. 2016).

References

Barnhart, Charles J., and Sally M. Benson. 2013. "On the importance of reducing the energetic and material demands of electrical energy storage". *Energy and Environmental Science* 6 (4). www.doi.org/10.1039/c3ee24040a.

Bauer, C., R. Bolliger, M. Tuchschmid, and M. Faist-Emmenegger. 2007. "Ecoinvent Report 6-VIII Wasserkraft". In *Sachbilanzen von Energiesystemen: Grundlagen für den ökologischen Vergleich von Energiesystemen und den Einbezug von Energiesystemen in Ökobilanzen für die Schweiz*, ed. by R. Dones, vol. 6-VIII. Dübendorf, Switzerland: Paul Scherer Institut, Swiss center for life cycle inventories.

Bogdanov, D., J. Farfan, K. Sadovskaia, A. Aghahosseini, M. Child, A. Gulagi, A. S. Oyewo, L. de Souza Noel Simas Barbosa, and C. Breyer. 2019. "Radical transformation pathway towards sustainable electricity via evolutionary steps". *Nat Commun* 10 (1): 1077. www.doi.org/10.1038/s41467-019-08855-1.

Brinkerink, Maarten, Brian Ó Gallachóir, and Paul Deane. 2021. "Building and Calibrating a Country-Level Detailed Global Electricity Model Based on Public Data". *Energy Strategy Reviews* 33. www.doi.org/10.1016/j.esr.2020.100592.

Brown, T., D. Schlachtberger, A. Kies, S. Schramm, and M. Greiner. 2018b. "Synergies of sector coupling and transmission reinforcement in a cost-optimised, highly renewable European energy system". *Energy* 160:720–739. www.doi.org/10.1016/j.energy.2018.06.222.

Crenna, Eleonora. 2022. *Preliminary life cycle inventory for LFP batteries.* Personal Communication.

Crenna, Eleonora, Marcel Gauch, Rolf Widmer, Patrick Wäger, and Roland Hischier. 2021. "Towards more flexibility and transparency in life cycle inventories for Lithium-ion batteries". *Resources, Conservation and Recycling* 170. www.doi.org/10.1016/j.resconrec.2021.105619.

Department of Energy. 2022. *Global energy storage database.* Web Page. https://sandia.gov/ess-ssl/gesdb/public/statistics.html.

Desing, Harald, Gregor Braun, and Roland Hischier. 2020. "Ecological resource availability: a method to estimate resource budgets for a sustainable economy". *Global Sustainability* 3:1–11. www.doi.org/10.1017/sus.2020.26.

Desing, Harald, and Rolf Widmer. 2021. "Reducing climate risks with fast and complete energy transitions: applying the precautionary principle to the Paris agreement". *Environmental Research Letters* 16 (12). www.doi.org/10.1088/1748-9326/ac36f9.

Grote, K.H., and J. Feldhusen. 2005. *Dubbel - Taschenbuch für den Maschinenbau.* 21st ed. Springer.

IPCC. 2018. *Global Warming of 1.5 degree C. An IPCC Special Report on the impacts of global warming of 1.5 degree C above pre-industrial levels and related global greenhouse gas emission pathways, in the context of strengthening the global response to the threat of climate change, sustainable development, and efforts to eradicate poverty.* Report. Intergovernmental Panel for Climate Change.

– . 2021. *Sixth Assessment Report: Physical Science Basis.* Report. IPCC. https://www.ipcc.ch/report/sixth-assessment-report-working-group-i/.

Koohi-Fayegh, S., and M. A. Rosen. 2020. "A review of energy storage types, applications and recent developments". *Journal of Energy Storage* 27. www.doi.org/10.1016/j.est.2019.101047.

Mutel, Chris. 2017. "Brightway: An open source framework for Life Cycle Assessment". *The Journal of Open Source Software* 2 (12). www.doi.org/10.21105/joss.00236.

Pleßmann, G., M. Erdmann, M. Hlusiak, and C. Breyer. 2014. "Global energy storage demand for a 100% renewable electricity supply". *Energy Procedia* 46:22–31. www.doi.org/10.1016/j.egypro.2014.01.154.

Sterner, Michael, and Michael Specht. 2021. "Power-to-Gas and Power-to-X—The History and Results of Developing a New Storage Concept". *Energies* 14 (20). www.doi.org/10.3390/en14206594.

Thema, M., F. Bauer, and M. Sterner. 2019. "Power-to-Gas: Electrolysis and methanation status review". *Renewable and Sustainable Energy Reviews* 112:775–787. www.doi.org/10.1016/j.rser.2019.06.030.

Wernet, Gregor, Christian Bauer, Bernhard Steubing, Jürgen Reinhard, Emilia Moreno-Ruiz, and Bo Weidema. 2016. "The ecoinvent database version 3 (part I): overview and methodology". *The International Journal of Life Cycle Assessment* 21 (9): 1218–1230. www.doi.org/10.1007/s11367-016-1087-8.

Zhang, Xiaojin, Christian Bauer, Christopher L. Mutel, and Kathrin Volkart. 2017. "Life Cycle Assessment of Power-to-Gas: Approaches, system variations and their environmental implications". *Applied Energy* 190:326–338. www.doi.org/10.1016/j.apenergy.2016.12.098.

Ziegler, Micah S., and Jessika E. Trancik. 2021. "Re-examining rates of lithium-ion battery technology improvement and cost decline". *Energy and Environmental Science* 14 (4): 1635–1651. www.doi.org/10.1039/d0ee02681f.
